# A Penny Learned: A Pilot Study on Financial Confidence and Wellness in Urban Community Hospital Psychiatrists

**DOI:** 10.1007/s40596-025-02147-1

**Published:** 2025-04-25

**Authors:** Yekaterina Angelova, Roaa Jambi, Marie Thearle, Chibuzo Ukonu, Omar Mirza

**Affiliations:** NYC Health + Hospitals/Harlem, New York, NY USA

**Keywords:** Financial literacy, Financial education, Physician wellness, Physician burnout

## Abstract

**Objective:**

This pilot study examines the perceived value of financial education and whether a brief didactic intervention improves financial confidence and sense of well-being for attending and resident psychiatrists.

**Methods:**

An anonymous survey was administered to attending and resident psychiatrists before and after a brief seminar on common financial topics. Aggregate data were analyzed using descriptive statistics. Unpaired *t*-tests were used to compare the pre- and post-seminar data due to anonymity of responses and high attrition rate.

**Results:**

Of the 36 participants (14 men and 22 women) who completed the pre-seminar survey, 7 (19%) had student loans, 30 (83%) had no prior financial education, 33 (92%) considered financial literacy valuable, and 28 (78%) believed that finances were related to wellness. Financial confidence was normally distributed and averaged 54% of the possible maximum. Men trended toward higher financial confidence (*p* = 0.06). Financial confidence was not associated with age, race, level of training, or debt. Burnout was generally low in this cohort. No significant differences in the perceived value of financial literacy, relationship between finances and wellness, or financial confidence before and after the financial seminar were identified (*p* = 0.37), though the post-seminar survey had only 14 (39%) respondents.

**Conclusions:**

Although the power of this study was insufficient to demonstrate an association between formal didactics and improvement in financial confidence and burnout, psychiatrists perceive significant value in such education. Findings additionally suggest possible gender differences in financial confidence, reflecting larger systemic inequities in physician financial wellness.

Financial literacy, the ability to understand and effectively use various financial skills, is crucial for financial well-being and financial independence, which have, in turn, been associated with reduction in burnout in physicians in training as well as practicing clinicians [1, 2]. However, studies consistently show that physicians receive little formal financial education and lack confidence in their financial literacy.

Survey studies in various physician groups (e.g., radiation oncologists, surgeons, emergency physicians) have identified low financial literacy among medical trainees and attending physicians [3–5]. A systematic review of financial curricula for medical trainees highlighted benefits including improved financial literacy, positive financial behavior, and improvement in participants’ well-being following implementation [6]. Another systematic review found that although very few publications on financial literacy and well-being actually describe financial curricula, those that did described beneficial effects on financial knowledge, decision-making, and well-being [7].

Some medical schools have implemented “mini-MBA” programs, providing education on personal finances, investing, and health care billing and payment models. Although the mini-MBA course was shown to improve financial knowledge and help participants feel more confident in managing their finances and planning for the future, such interventions can be time-consuming and costly and do not address the needs of physicians who have completed their medical training [8]. There remains no clear direction for a standard intervention to address physicians’ financial education needs.

Literature on financial education, literacy, and confidence in psychiatrists specifically is scarce. Furthermore, most prior investigations of this topic focus on US medical graduates. The purpose of this study is to examine current perception of the value of financial education and to evaluate whether a brief financial didactic intervention improves confidence and perceived sense of well-being at an urban community hospital.

## Methods

An anonymous electronic survey was administered to a convenience sample of attending and resident psychiatrists before and after a brief educational seminar series addressing common finance-related topics. Three seminars were conducted free of charge by a reputable financial advising service that provides nationwide educational events for physicians at different stages in their careers. The first seminar addressed the basics of money management (e.g., budgeting, savings, strategies for optimizing personal cash flow) and strategies for assessing one’s current financial situation and setting financial goals. The second seminar focused on management of student loan debt (e.g., potential repayment and loan forgiveness options). The third seminar overviewed basic investment principles and tackled common questions about disability insurance, life insurance, and retirement planning. Each lasted 1 h, with 45 min of didactic material and 15 min for questions. Attendance ranged from 25 to 30 individuals per seminar. Participation was voluntary.

All resident and attending physicians in the department of psychiatry were invited by email to participate in the seminars and surveys. Because the study was of less than minimal risk, the institutional review board determined that written informed consent could be waived.

The pre-seminar survey and post-seminar survey consisted of demographic questions pertaining to participants’ age group, identified gender, race/ethnicity, and current level of training or years in clinical practice. Participants were asked to comment on their current financial situation, including financial situations that apply to them (e.g., presence of debt, student loans, home ownership, investment, business ownership), the presence of any financial dependents (defined as someone who relies on another for financial support; examples provided to respondents included children and parents), and frequency of engagement with finances (e.g., checking a balance, buying a stock).

Participants were asked about any prior financial education during their medical training or faculty development and their perceived value of financial literacy, using a Likert scale ranging from 1 to 5, with 1 being not at all valuable and 5 being very valuable. These questions were followed by nine questions related to the participants’ current financial “mood” via Likert scales (1 = not at all, 5 = very much) on participants’ perceived understanding of their personal finances, financial goals for the future, worries related to finances, confidence in managing debt, budgeting and saving money, confidence in asset protection strategies, retirement planning and investment strategies, and knowledge of where to turn for financial advice if needed. These questions were developed based on the proposed seminar topics and issues relevant to physicians. Finally, participants were asked to comment on how much they associate finances with their overall well-being and rate their current level of burnout on a single-item burnout measure, a valid substitute for the Maslach Burnout Inventory emotional exhaustion (MBI:EE) [9, 10]. The single-item measure was utilized to reduce the time burden of the surveys and thus increase the likelihood of survey completion.

Aggregate data from pre-seminar and post-seminar surveys were analyzed to detect changes in perceived financial literacy, confidence, and burnout level before and after the educational series. A summary measure of financial confidence was created by adding together scores (each normalized on a scale from 1 to 5) from the nine financial “mood” questions. One question assessing the perceived value of financial education and its importance to wellness was excluded from this sum. The potential minimum score for this measure was 9, indicating low financial confidence, and the maximum was 45, indicating high confidence.

Continuous data are reported as mean ± standard deviation and categorical data as proportions. Differences in variables between groups were compared using Student’s *t*-tests and 1-way ANOVA for Likert scales and Chi-square tests for categorical data. A factor analysis was done to confirm the structure of the nine items of the financial confidence measure with calculation of eigenvalues. The internal consistency (reliability) of the construct was assessed by calculating the coefficient (Cronbach’s) alpha. Unpaired *t*-tests were used to compare the anonymous pre-seminar and post-seminar data as surveys were submitted anonymously.

## Results

From the 53 individuals invited to the seminars (26 attendings, 27 residents), 36 (68%) participants (16 (44%) attendings, 20 (56%) residents) completed the pre-seminar survey, and 14 (39%) participants (7 attendings, 7 residents) completed the post-seminar survey. Of note, all the invited residents were international medical graduates. Roughly 60% of participants in both surveys identified as women (*n* = 22 pre-seminar). Among participants, 21 (58%) were 30–40 years old and the rest were older than 40. Various training levels and roles were represented, although the highest participation was among attending physicians and postgraduate-year 4 (PGY-4) residents (*n* = 23 (64%)). The study captured a racially and ethnically diverse population, with 14 (39%) identifying as Asian or Pacific Islander and 7 (19%) identifying as Black or African American. Twice as many participants had a dependent than participants with none.

**Fig. 1:**
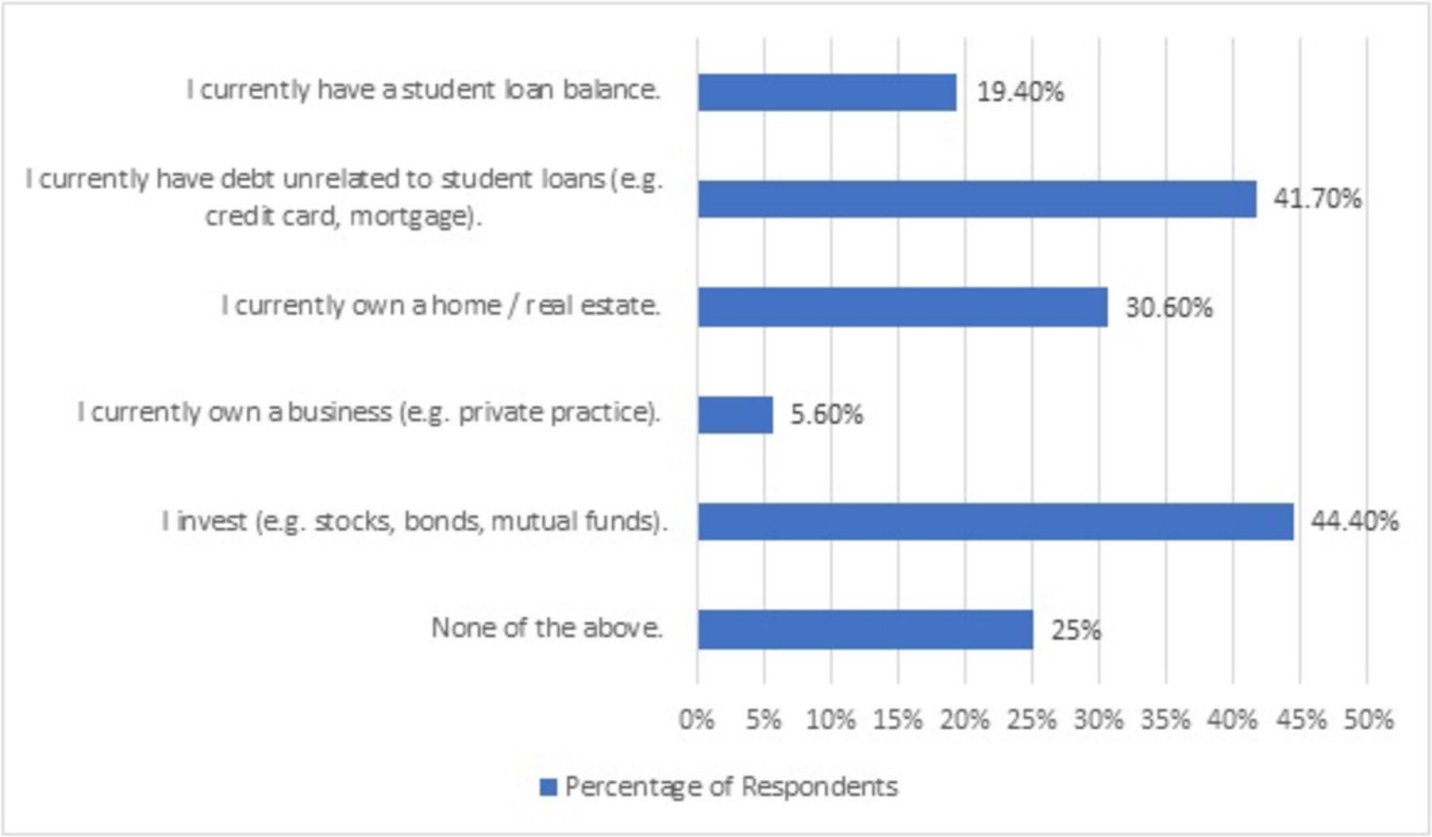
Current financial situations reported by respondents in the pre-seminar survey

Of the 36 participants in the pre-seminar survey, 7 (19%) had student loans (4 attendings, 3 residents), 15 (42%) had other debt, and 16 (44%) reported investing, with additional common financial situations reported in Fig. 1. Most (*n* = 27 (75%)) participants reported weekly or monthly engagement with their finances (e.g., checking balances, reading finance blogs, buying stocks); 3 (9%) engaged with their finances daily; and 4 (11%) reported no more than annual engagement.

Five participants (14%) reported prior formal financial education during medical training or faculty development, while 30 (83%) had no prior financial education and 1 (3%) did not recall any. Responses highlighted participants’ appreciation of the need for improved financial literacy and competence, with 33 (92%) selecting a high (4 or 5) rating on the Likert scale. Twenty-three (64%) reported high or very high levels of worry and no one reported having no worry at all.

**Fig. 2:**
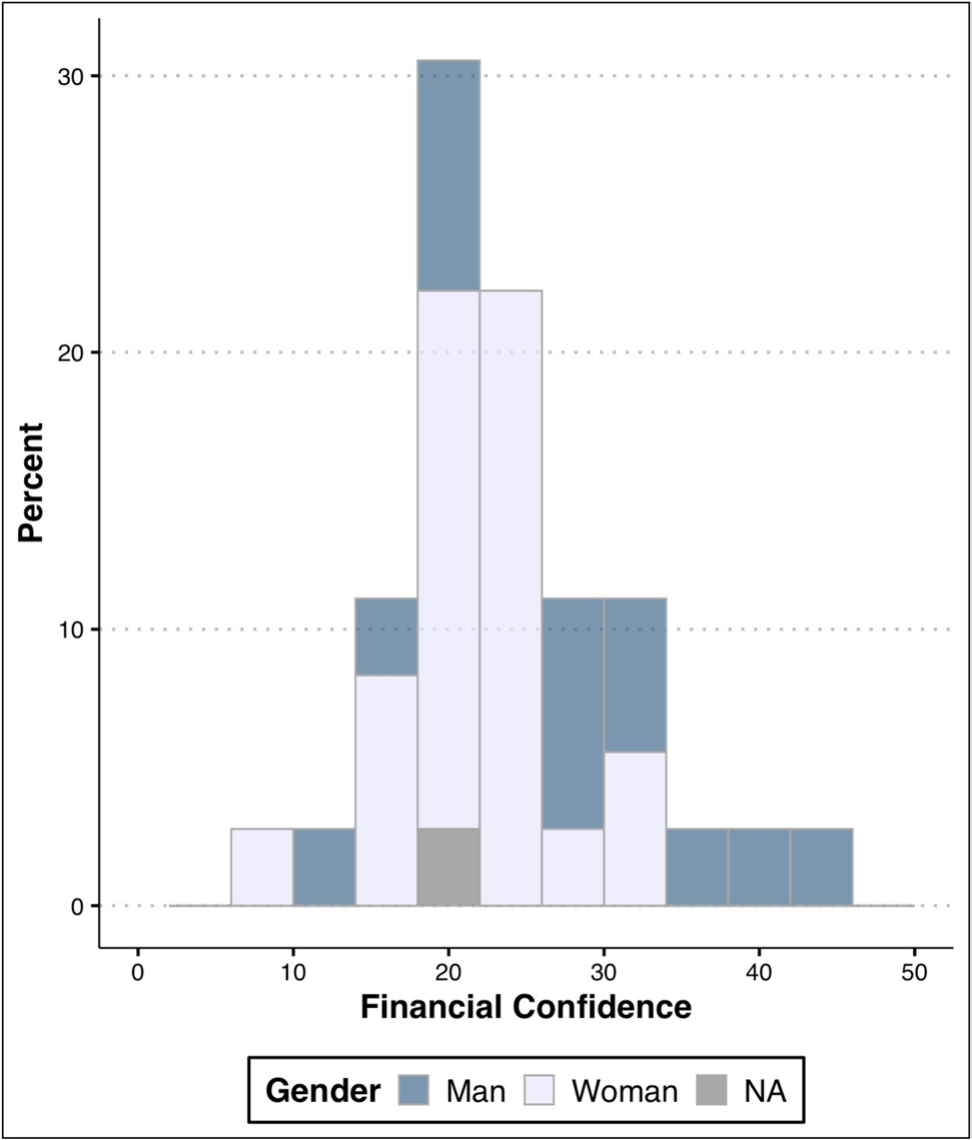
Histogram of financial confidence measure scores among pre-seminar survey respondents by gender

Eigenvalues confirmed the financial confidence summary measure was unidimensional. The coefficient alpha (= 0.89 [95% CI 0.83, 0.94]) indicated good internal consistency. Financial confidence averaged only 54% of the possible maximum. The most strongly associated items with the financial confidence construct were confidence in protecting assets, arranging a retirement plan, and understanding investment strategies. Financial confidence scores ranged between 10 and 44 and were normally distributed, with a mean of 24.4±7.3 (Fig. 2). Men trended toward a higher overall financial confidence (27.5±9.3 compared to 22.7±5.5 for women, *p* = 0.06). Financial confidence did not differ between attendings and residents (24.8 ± 7.9 vs 24.2 ± 7.0; *p* = 0.81). Having a dependent was most strongly associated with financial confidence, with participants who reported having dependents (*n* = 23) possessing a higher financial confidence of 27.1±7.0 compared to 19.2±5.2 for those without dependents (*n* = 12; *p* = 0.0016). Financial confidence was not associated with age or race, nor was there an association between debt and financial confidence or lack thereof (*p* = 0.19).

In the pre-seminar survey, 28 (78%) stated that finances were very much associated with their wellness (4 to 5 on Likert scale). Burnout was generally low among participants, with 26 (72%) reporting mild or no symptoms on the Single Item Burnout Measure. PGY-4 residents reported the lowest burnout scores, with all 7 (100%) reporting a 1 or 2 on the Likert scale.

The study identified no significant difference between pre-seminar and post-seminar scores related to perceived value of financial literacy (average score of 4.7 on both surveys), the perceived relationship between finances and general wellness (average score of 4 on both surveys), or overall financial confidence (scores of 24.4±7.3 on the pre-seminar survey and 26.4±6.3 on the post-seminar survey; *p* = 0.37).

## Discussion

In this study, few psychiatrists reported encountering formal financial education during medical training, but most felt that financial literacy is valuable and that overall wellness is related to finances. Debt was not associated with financial confidence, though having a dependent was, possibly indicating that the added responsibility of caring for a dependent may have influenced personal efforts to increase financial knowledge. This study also found that financial confidence did not increase with age or level of clinical training or role, suggesting that life experience alone may not be sufficient to improve financial confidence. As such, formal financial curriculum is crucial in medical training and faculty development. Compared to a recent meta-analysis [11] estimating significant burnout in a quarter to half of psychiatrists, this sample reported burnout rate on the lower end of this range.

At baseline, participants had only moderate confidence in their financial aptitude, despite potential for a high income and highly rating the need for financial literacy. Women trended toward a lower financial confidence, which may be a partial explanation for the higher proportion of women participating in the seminar series. The gender gap in financial literacy is well known, as is the wage gap between men and women [12]. Although residency salaries are standardized, protecting from wage gaps, significant wage gaps favor male psychiatry attendings. Male psychiatrists were earning about 21% more than their female peers in 2020 and 23% more in 2022 [13, 14]. These gender gaps may be directly related to financial confidence, highlighting the need for financial education. Given the time requirements of medical training and practice, this would need to be formally incorporated into curriculums. Alternatively, lower financial confidence in women may be a result of structural inequities resulting in wage gaps, reflecting a larger systemic issue.

Recent studies show that US medical school graduates average over $240,000 in total student loan debt [15]. This debt was found to be associated with personal life dissatisfaction, career regret, and burnout [16]. Our cohort is relatively unique in that all the residents were medical graduates from outside the USA, where medical education often results in less debt. This may have contributed to the relatively low burnout observed in our cohort. Unfortunately, the attendings’ medical schools were unknown.

This study found no significant difference in perceived value of financial literacy, effect of financial education on overall wellness, or financial confidence before and after a brief didactic series on select financial topics. Due to the anonymity of the surveys, an unpaired *t*-test was used for statistical analysis; a paired test would have more power to detect a statistical difference following the brief didactic intervention, if one exists.

Significant limitations include the small sample size and attrition between the pre- and post-surveys. The low post-seminar rate may be attributed to factors such as physicians’ competing responsibilities (e.g., clinical, administrative, and supervisory duties), which may have led participants to deprioritize completion of the post-survey. Additionally, the surveys asked similar questions, which may have led respondents to only complete the first, if they felt their answers had not changed.

Our study may not be generalizable to physicians with large amounts of educational debt. Also, due to the anonymous nature of the survey in a small sample, we did not ask residents about future career plans and, thus, were unable to determine how debt load might influence career choice. Similarly, we did not explore the potential impact of the financial situations of respondents’ spouses and families on financial confidence and worry. Finally, the post-seminar survey did not explore respondents’ perception of the seminars, including satisfaction with the quality and content of the seminars or any resulting confusion or anxiety.

We conceptualized financial confidence as individuals’ perceived understanding and comfort with their personal financial situation and various financial topics. We did not measure financial literacy. Our financial confidence measure had good internal consistency within the population of this pilot study but will need to be confirmed in future studies.

Standardized and widespread financial curriculum for medical trainees and practicing physicians may be an essential step toward financial confidence and physician well-being. A brief, high-yield curriculum such as in this study can be seamlessly incorporated into routine faculty experiences such as grand rounds, conferences, and faculty development activities. Faculty wellness programs can also provide access to educational resources pertaining to finance to maximize physician well-being. Resources from national organizations also could promote further standardization and ongoing evaluation of financial education in medical training and faculty development.

## Data Availability

Anonymous aggregate dataset available upon reasonable request.
